# Understanding the Integrated Health Management System Policy in China From Multiple Perspectives: Systematic Review and Content Analysis

**DOI:** 10.2196/47197

**Published:** 2024-01-24

**Authors:** Yang Yu, Sufen Wang, Lijue You

**Affiliations:** 1 Glorious Sun School of Business and Management Donghua University Shanghai China; 2 Department of Informatics Huadong Hospital Affiliated to Fudan University Shanghai China

**Keywords:** integrated health management system, medical association, medical consortium, policy tools, content analysis, PRISMA, Preferred Reporting Items for Systematic Reviews and Meta-Analyses

## Abstract

**Background:**

The integrated health management system (IHMS), which unites all health care–related institutions under a health-centered organizational framework, is of great significance to China in promoting the hierarchical treatment system and improving the new health care reform. China’s IHMS policy consists of multiple policies at different levels and at different times; however, there is a lack of comprehensive interpretation and analysis of these policies, which is not conducive to the further development of the IHMS in China.

**Objective:**

This study aims to comprehensively analyze and understand the characteristics, development, and evolution of China’s IHMS policy to inform the design and improvement of the system.

**Methods:**

We followed the PRISMA (Preferred Reporting Items for Systematic Reviews and Meta-Analyses) guidelines to collect 152 policy documents. With the perspective of policy tools and policy orientation as the core, a comprehensive 6D framework including policy level, policy nature, release time, policy tools, stakeholders, and policy orientation was established by combining the content of policy texts. These dimensions were then analyzed using content analysis.

**Results:**

First, we found that, regarding the coordination of policy tools and stakeholders, China’s IHMS policy was more inclined to use environment-based policy tools (1089/1929, 56.45%), which suggests a need for further balance in the internal structure of policy tools. Attention to different actors varied, and the participation of physicians and residents needs further improvement (65/2019, 3.22% and 11/2019, 0.54%, respectively). Second, in terms of level differences, Shanghai’s IHMS policy used fewer demand-based policy tools (43/483, 8.9%), whereas the national IHMS policy and those of other provinces and cities used fewer supply-based tools (61/357, 17.1% and 248/357, 69.5%, respectively). The national IHMS strategy placed more emphasis on the construction of *smart health care* (including digital health; 10/275, 3.6%), whereas Shanghai was a leader in the development of *healthy community* and *healthy China* (9/158, 5.7% and 4/158, 2.5%, respectively). Third, in terms of time evolution, the various policy tools showed an increasing and then decreasing trend from 2014 to 2021, with relatively more use of environment-based policy tools and less use of demand-based policy tools in the last 3 years. The growth of China’s IHMS policy can be divided into 3 stages: the *disease-centered period* (2014-2017), the *e-health technology development period* (2017-2019), and the *health-centered period* (2018-2021).

**Conclusions:**

Policy makers should make several adjustments, such as coordinating policy tools and the uneven relationships among stakeholders; grasping key policy priorities in the context of local characteristics; and focusing on horizontal, multidimensional integration of health resources starting from the community. This study expands the objects of policy research and improves the framework for policy analysis. The findings provide some possible lessons for future policy formulation and optimization.

## Introduction

### Background

The integrated health management system (IHMS) is a form of integrated medical care in China that has gained significant attention in recent years. The World Health Organization defined integrated medical care in its 1996 report as “integrating the management and organization of services, providing residents with health services that are easy to access and willing to accept, so that the expenditure on health services can be controlled, and the final service effect can achieve the expected health benefits” [1]. The IHMS is a health management consortium that places health at the center of its operations. Its core service content focuses on “preventing serious diseases, managing chronic diseases, and promoting health” [2]. Medical and health service institutions are the running subject of IHMS, and it aims to address the problem of unreasonable and uneven distribution of medical resources while meeting the rapidly rising demand for various medical and health services, from disease prevention to postoperative care. Compared with medical associations, the IHMS has a wider range of organizations, emphasizing disease prevention and classified management and expanding the functions and services of medical associations [2]. With the promulgation of *Healthy China 2030* and *Healthy China Action (2019-2030),* China has started to transition from a *treatment-centered* approach to a *health-centered* one [3].

Existing research on IHMS is mainly carried out from 2 perspectives. The first perspective explores IHMS by analyzing all aspects of integrated medical operation. Early literature limited it to vulnerable groups such as older adults or patients with chronic diseases, cancers, and other diseases [4]. However, with the increasing attention paid to the health rights of individuals throughout their life cycle, the clients of integrated medical care have expanded to encompass the entire population [4]. Integration types can be divided into functional integration [5], professional integration [6], normative integration [7], and system integration [8]. Functional integration refers to the coordination between different health care activities and support functions. Professional integration, in contrast, aims to create synergy and integration between health care providers whether they work within the same organization or in different ones. Normative integration aims to achieve an agreement on the normative aspects among all stakeholders involved in health care integration. It also aims to ensure timely reflection on all relevant changes brought about by integration and collaboration in the provision of health services while reinforcing interdependence and collective responsibility. Systemic integration means looking at the complexity of health care organizations in a holistic context and considering the interrelationships between the stakeholders involved in the integration process and the overall context. The direction of integration is divided into horizontal and vertical integration [9]. Horizontal integration refers to the linking of similar services and organizations at the same level to optimize the supply structure, share technical equipment and services, or reduce costs through collective purchasing, for example, horizontal integration between general practitioners and organizations such as community pharmacies and community hospitals in the United Kingdom. Vertical integration refers to the combination of organizations at different levels under a unified management system to form a scale of health care services to diversify risks and reduce the cost of purchasing health care products while providing seamless services to patients, such as the close-knit county medical communities being implemented across China. In terms of the evaluation of integration effects, scholars have put forward various models. Berwick et al [10] put forward a 3-objective model according to the integration practice in the United States. Sikka et al [11] then improved it and developed a new 4-quadrant model. In the same year, Nurjono et al [12] synthesized dozens of key characteristic indexes, further revised them using the Delphi method, and put forward the Rainbow Model. In 2019, Chaitkin et al [13] proposed a 5-step decision-making theoretical framework. With the development of big data, the internet of things, and other technologies, recent research on integrated medical care is keen to explore the integration of e-medical, telemedicine, and integrated medical care. For example, Tossaint-Schoenmakers et al [14] used the structure-process-outcome framework of the Donabedian model to determine the relevant indicators for integrating eHealth into medical care organizations. Fatoum et al [15] explored the future impact of blockchain technology and its integration with other innovations in the health care ecosystem. de Luca et al [16] designed a comprehensive digital hypertension method to enable citizens to learn how to prevent the development of hypertension and lead a healthy lifestyle. These studies cover the concept, key elements, evaluation methods, and integration of cutting-edge technologies of integrated medical care, but the development of integrated medical care is often related to the development of relevant policy systems.

The second perspective pertains to the policy system related to the IHMS. In China, most of the existing research still centers on medical associations, with few studies on IHMS. There are primarily 3 types of research related to medical association policy. The first is to analyze the current situation by sorting out medical association policy. For instance, Ling et al [17] summarized the development and practice of medical association policy in China, identified issues such as weak 2-way referrals, and provided relevant suggestions. Although they carried out an in-depth qualitative analysis of the policy, they did not statistically analyze its textual content, meaning that, although they made a number of recommendations, these needed to be supported by more comprehensive quantitative data that would have helped provide deeper insights into their recommendations. The second type of research evaluates the implementation effect of medical association policy. For example, Yang et al [18] used a binary logistic regression model to analyze the implementation effect and influencing factors of medical association policy in Guizhou province and proposed measures such as increasing social health expenditure. Although this research is based on models, the data primarily come from field interviews or questionnaires, neglecting information in policy texts. The third type of research involves multidimensional analysis of policies regarding policy tools. For instance, Chen et al [19] conducted a statistical analysis of medical association policy issued by the central government from the perspective of policy tools and integrated medical care, concluding that the structure requires further adjustment. This type of research tends to be clearer and more operational as the dimensions and categories used are clearly identifiable, allowing the researcher to make judgments and categorize the research based on the definitions of each category, reducing the interference of subjective judgments, and improving the objectivity and reproducibility of the research. However, the number of policy texts they collected and the dimensions of the framework they constructed are relatively limited. In addition, the few studies on the IHMS policy are still in the concept innovation, concept definition, and case analysis phases, such as the studies by Bai [20] and Xiong et al [3].

### Objectives

The aim of this study was to comprehensively analyze China’s IHMS policy and provide accurate and comprehensive suggestions for policy optimization through quantitative data analysis, such as improving policy level and implementation, strengthening smart medical care and healthy community support, optimizing policy tools, strengthening stakeholder cooperation, and improving policy orientation so as to promote the effective implementation and development of the IHMS in China. On the basis of the contents and objectives of the relevant policy and planning documents issued by the Chinese government, we proposed the concept of China’s IHMS policy system, which refers to a set of health-centered policies that integrate various types of health care resources. The policy system covers a variety of policies (Multimedia Appendix 1) on healthy China; graded diagnosis and treatment; medical association; healthy community; and smart health care, which includes digital health.

The collection of these policies expands the number, scope, and subjects of China’s IHMS policy, constituting a policy chain–policy cluster–policy network health consortium policy system with common goals, multiple subjects, and numerous means, spanning levels and complex interactions. It covers various aspects of China’s IHMS policy, including health objectives, health service patterns, the integration of health resources, the application of IT, and community health services. Multiple policy objectives are unified under a holistic framework, and different policy orientations are organically combined to support and synergize each other to promote the development of health care.

On the basis of the researchers’ in-depth investigation and analysis of China’s IHMS policy, a 6D framework (Multimedia Appendix 2) was constructed, including policy level, policy nature, release time, policy tools, stakeholders, and policy orientation. Although it fully considers the characteristics of China’s IHMS policy, the framework exploits and effectively integrates the meta-information and key elements in the policy text and provides a structured analytical method that allows researchers to systematically conduct quantitative analysis and policy comparison. The framework focuses on policy tools and policy orientations, exploring their characteristics and trends under the dimensions of policy level, policy nature, and release time. Through this comprehensive analytical framework, we expect to provide policy makers and relevant researchers with more operational and practical methods for policy analysis.

This study focused on two main issues:

1. What are the release time, release unit, nature, and specifications of China’s IHMS policy? What types of policies, policy tools, and stakeholders are included?
2. With a focus on the 2 core dimensions of policy tools and policy orientation, what are the differences in the distribution of China’s IHMS policy in terms of policy level, policy nature, and policy release time?

By answering the aforementioned questions, this study aimed to explore whether the structure of policy tools and policy orientation of China’s IHMS is balanced, whether it changes with time, and what the similarities and differences are between local and central governments. The results of this study will aid in developing suggestions for policy optimization.

## Methods

### Overview

This study used a systematic literature review method [21] and a content analysis approach. First, policy documents related to the IHMS were collected according to the PRISMA (Preferred Reporting Items for Systematic Reviews and Meta-Analyses) criteria and then sorted by release time and formulation subject, among other criteria. Finally, they were organized, coded, and analyzed using the 6D framework based on policy tool theory.

### Research Materials

#### Overview

The research materials were obtained from various sources, and a systematic review was conducted with the following objectives in mind: (1) being relevant to the background of new medical reform or integrated medical care; (2) involving one or more aspects of health management or disease prevention, treatment, and care; and (3) paying attention to, advocating for, or promoting the development of new technologies in the medical field.

By thoroughly considering these objectives, we compiled a collection of highly relevant and diverse IHMS policy literature. The PRISMA checklist is shown in Multimedia Appendix 3.

#### Information Sources

We searched for relevant articles in the following databases: Chinese People’s Government Network, China National Health Commission Network, China Provincial People’s Government Network, China Provincial Health Commission Network, and Bailu think tank. These databases have high authority, comprehensive policy literature, clear classification, and convenient access, making them suitable for this research.

#### Selection Criteria

To select the literature for analysis, a number of inclusion and exclusion criteria were used, as shown in Textbox 1.

**Inclusion and exclusion criteria for the collection of research materials.**

**Inclusion criteria**

- Article type: public documents, bulletins, and notices
- Article language: Chinese articles
- Article publication time: articles published before the end of 2021
- Article main content: 5 categories of policies as identified by the definition of integrated health management system (IHMS) policies in China

**Exclusion criteria**

- Article type: other types of articles, such as media reports and policy interpretations
- Article language: articles in other languages
- Article publication time: articles that had not been published by the study launch time
- Article main content: articles not directly related to the definition of China’s IHMS policy

#### Screening of Research Materials

**Figure 1:**
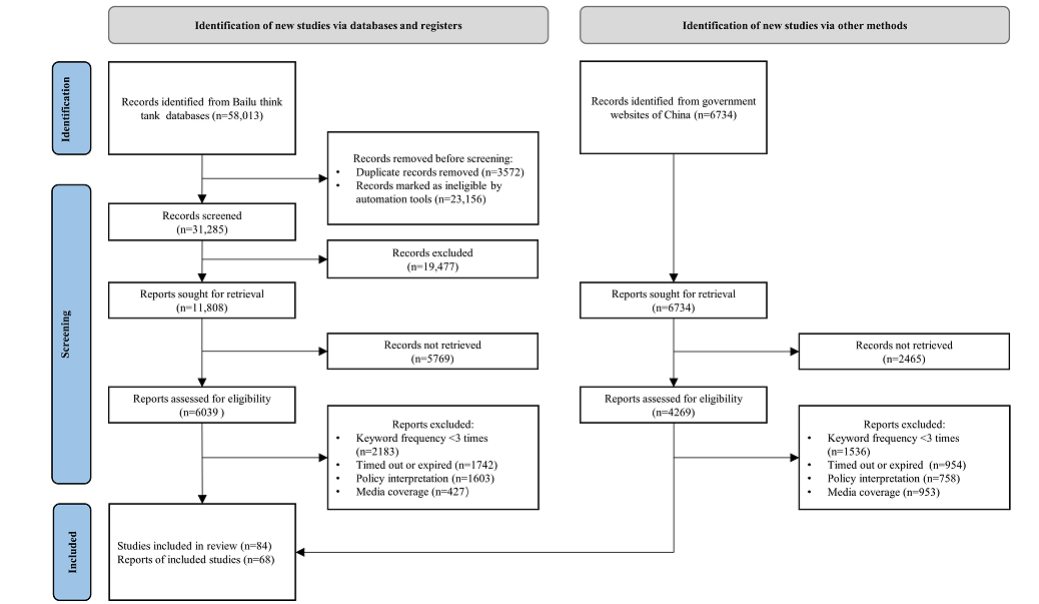
Search process according to the PRISMA (Preferred Reporting Items for Systematic Reviews and Meta-Analyses) criteria.

The process of searching for and selecting policy documents was divided into four stages, as outlined in Figure 1:

1. Keyword determination: articles were searched for in electronic databases using the keywords *healthy China*, *graded diagnosis and treatment*, *medical association*, *healthy community*, and *smart health care*.
2. Preliminary review: identified articles were sorted by relevance, and titles and introduction contents were reviewed to select important documents according to the selection criteria.
3. Further review: articles that passed the preliminary review were read.
4. Material determination: articles with a high number of citations were browsed to obtain more research materials.

As a result, a total of 152 policy documents, including plans, outlines, and notices, were obtained, covering a time span from July 2014 to August 2021. Multimedia Appendix 4 provides a complete list.

### Research Framework

To ensure that the theoretical framework was rigorous and practical, we conducted in-depth analyses of practical application scenarios. From the perspective of practical application, the current policy analysis in the industry focuses on the following three key aspects: (1) policy specifications and nature, with particular emphasis on policies issued by the central government; (2) policy synergies, that is, whether complementary policies and measures are sufficient; and (3) whether the policies can achieve the expected results, which is also the final point of the policy analysis. In addition, unlike general single-category policies, the IHMS policy is a policy chain–policy cluster–policy network system formed by multicategory policies. The policy orientation, that is, the main elements of the policy objectives, will play a very important role in the implementation effect of the entire system.

However, the 2D or 3D analytical frameworks mainly used in existing studies have limitations in these important aspects. For example, Tang and Si [22] did not make full use of the metainformation of policy documents, such as the release time, issuing agency, and policy type, although they conducted a joint analysis of policy tools and stakeholders, and Shen et al [23], although they conducted an external characterization analysis that included policy year, issuing agency, and policy type and also conducted a joint analysis of policy tools and health systems, did not address the differences in the external characterization dimensions of policy tools. The insufficient exploration of policy synergies limits the scope and diversity of policy analyses.

**Figure 2:**
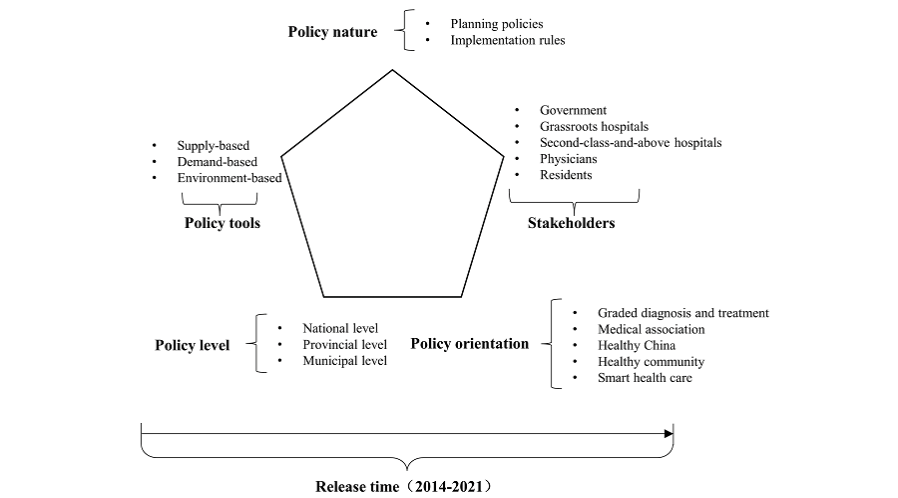
6D analysis framework for China’s integrated health management system policy.

Therefore, in addition to policy tools and stakeholders, this study added the 4 dimensions of policy level, policy nature, release time, and policy orientation. These dimensions are interrelated and form an overall analytical framework structure, as shown in Figure 2. Policy level, policy nature, release time, and policy orientation are vertical dimensions, indicating the different characteristics of policies. Policy tools and stakeholders are horizontal dimensions, indicating the tools and relevant stakeholders in policy implementation. Meanwhile, policy orientation, as a specific dimension, runs through the entire analytical framework, reflecting the importance of policy objectives.

This study adopted the policy tool theory by Rothwell and Zegveld [24] to define 3 types of IHMS policy tools based on the characteristics of the IHMS (Table S1 in Multimedia Appendix 5). Supply-based tools mainly refer to policies that provide direct support for the IHMS, such as funding, talent, technology, and other resources [25]. Demand-based tools mainly refer to policies that promote the IHMS by creating a “pull” effect, such as encouraging the public to use health care services through improved service quality and accessibility. Environment-based tools indirectly promote the IHMS by creating an enabling environment through the establishment of goals, plans, incentives, and other policy measures. By analyzing the use of these policy tools in IHMS policies, we can gain insights into the policy-making strategies and priorities of relevant government departments.

Furthermore, the implementation of the IHMS involves various stakeholders, each with different roles to play. To account for this, we added a stakeholder dimension to create a 2D analysis framework for IHMS policy texts. The stakeholders of the IHMS include the government, grassroots hospitals, second-class-and-above hospitals, physicians, and residents. The government leads and formulates IHMS policies, whereas grassroots hospitals and second-class-and-above hospitals are key players in implementing the IHMS and providing health services to residents through their collaboration. Physicians at all levels serve as the backbone of the IHMS, whereas residents are the direct beneficiaries of the policy [22]. Table S2 in Multimedia Appendix 5 displays the categories and definitions of the stakeholders.

### Coding and Statistics

Using the paragraph symbol as a separator, the 152 policy documents obtained from the screening were segmented into a collection of text paragraphs, retaining information such as the title, author, and date of each document but excluding additional tables. We ended up with 1345 policy texts as research material.

We coded the release time, policy level, policy nature, and policy orientation based on the year in which the policy was issued, the administrative level to which the policy-issuing organization belongs, the title and its lineage, and the 5 categories of policies that make up the IHMS. For the policy tools and stakeholders, coding was based on the categorization of policy tools in Table S1 in Multimedia Appendix 5 and the categorization of stakeholders in Table S2 in Multimedia Appendix 5. For example, for the following policy text—“promoting the construction of public health system in medical complexes. Hospitals...”—the policy tools can be coded as *functional localization*, *technical support*, and *talent construction*, whereas stakeholders can be coded as *government*, *grassroots hospitals*, and *second-class and above hospitals*.

Multimedia Appendix 6 shows the specific steps for coding and manual labeling.

We coded the policy texts using Microsoft Excel (Microsoft Corp) and calculated an average κ coefficient value of 74.6%, indicating a high degree of consistency (substantial) for the 2 coders under each dimension of categorization using SPSS (IBM Corp). Following these steps, we ensured that the credibility of the manual annotation results was supported by independent annotation, comparative positioning, and discussion of the changes. This systematic approach to annotation helped reduce subjective interference and improve the accuracy and consistency of annotation, thereby increasing the reliability of the findings and conclusions.

## Results

### 1D Analysis Under the 6D Framework

#### Policy Release Time

**Figure 3:**
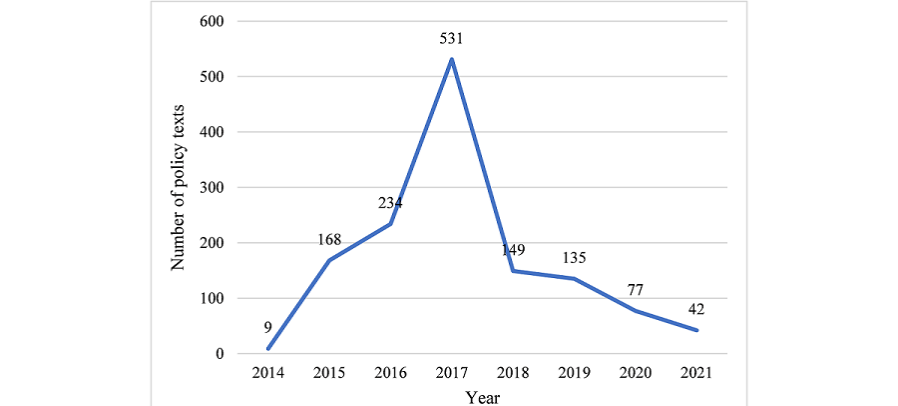
Time-varying chart of marked integrated health management system policy texts.

Figure 3 shows the distribution of the IHMS policy release time. The number of IHMS policy texts showed an overall upward trend from 2014 to 2021. This number increased rapidly before 2017, reaching a peak of 531 that year. However, in recent years (not excluding the influence of the statistical period until August 2021), this number declined.

#### Policy Level

The distribution of the IHMS policy levels is presented in Table 1. The data revealed that there were 659 policy texts at the provincial level followed by 275 at the national level. In addition, there were >150 policy texts at other municipal levels as well as in Shanghai.

**Table 1:** Distribution of policy level of marked integrated health management system policy texts.

Policy level	Policies (n=1345), n (%)
Provincial level	659 (49)
National level	275 (20.45)
Municipal level	253 (18.81)
Shanghai	158 (11.75)

#### Nature of Policy

Table 2 shows the distribution of the policy nature of the IHMS. The number of planning and implementation rule policy texts was equivalent, at 674 and 671, respectively.

**Table 2:** Distribution of policy nature of marked integrated health management system policy texts.

Policy nature	Policies (n=1345), n (%)
Planning policy	674 (50.11)
Detailed regulations	671 (49.89)

#### Policy Tools

The distribution of the IHMS policy tools is presented in Table 3. Environment-based policy tools were the most frequently used, accounting for 56.45% (1089/1929) of all tools, followed by demand-based policy tools at 25.04% (483/1929), whereas supply-based policy tools were the least commonly used, accounting for only 18.51% (357/1929). This reflects the current situation, in which demand-based policy tools are relatively abundant, whereas supply-based policy tools are relatively scarce in China’s IHMS policy system.

**Table 3:** Distribution of policy tools of the integrated health management system (n=1929).

Policy tool and secondary classification		Policy tools, n (%)
**Supply-based (n=357)**		
	Talent construction	75 (3.89)
	Financial guarantee	37 (1.92)
	Information support	127 (6.58)
	Technical support	18 (0.93)
	Rational layout	100 (5.18)
**Demand-based (n=483)**		
	Medical insurance payment	73 (3.78)
	Medicine supply	40 (2.07)
	Price adjustment	38 (1.97)
	Ability building	59 (3.06)
	Adhere to public welfare	24 (1.24)
	Orderly medical treatment	197 (10.21)
	Demonstration project	52 (2.7)
**Environment-based (n=1089)**		
	Objective programming	223 (11.56)
	Institutional building	253 (13.12)
	Incentive and restraint	187 (9.69)
	Policy promotion	49 (2.54)
	Functional localization	241 (12.49)
	Resource sharing	136 (7.05)

Examining the specific types of policy tools, it was observed that, among environment-based policy tools, *institutional building*, *functional localization*, and *objective programming* were the most frequently used, totaling 37.17% (717/1929), whereas *policy promotion* was used the least, accounting for only 2.54% (49/1929). Among the demand-based policy tools, only *orderly medical treatment* was widely used, and the frequency of use of *adhere to public welfare*, *medicine supply*, and *price adjustment* was <2.5% (24/1929, 1.24%; 40/1929, 2.07%; 38/1929, 1.97%, respectively). The proportion of supply-based policy tools was also relatively small, with the most commonly used tool, *information support*, accounting for only 6.58% (127/1929), whereas *technical support* and *financial guarantee* were the least frequently used, accounting for 2.85% (55/1929) in total.

#### Stakeholders

Table 4 presents the distribution of stakeholders in IHMS policy. The highest frequency was of governments at 57.16% (1154/2019), followed by *second-class and above hospitals* and *grassroots hospitals* accounting for 39.08% (789/2019) in total. *Residents* had the lowest participation frequency at only 0.54% (11/2019). This indicates that the government plays a leading role in the entire IHMS system, and general and grassroots hospitals at or above the second level play a significant role as the main service providers. However, the policy pays relatively little attention to family physicians, specialists, and residents.

**Table 4:** Distribution of stakeholders in the integrated health management system (n=2019).

Stakeholders	Participation, n (%)
Governments	1154 (57.16)
Grassroots hospitals	461 (22.83)
Second-class-and-above hospitals	328 (16.25)
Physicians	65 (3.22)
Residents	11 (0.54)

#### Policy Orientation

Table 5 displays the distribution of IHMS policy orientation. Medical associations accounted for the highest percentage with 778 policy texts, representing 57.84% (778/1345) of the total. Graded diagnosis and treatment orientation was the second most common with 486 copies, accounting for 36.13% (486/1345). In contrast, smart health care, healthy community, and healthy China had <50 policy texts each. Compared with medical associations and graded diagnosis and treatment, the existing IHMS policy framework covers relatively little content on smart health care, healthy community, and healthy China. However, it is noteworthy that healthy China is a strategic policy orientation, and the related contents are mainly top-level design plans. The specific measures were refined into medical associations, graded diagnosis and treatment, smart health care, healthy community, and other aspects. Therefore, the number of policy texts in this category was the lowest.

**Table 5:** Distribution of policy orientation of marked integrated health management system policy texts.

Policy orientation	Policies (n=1345), n (%)
Medical association	778 (57.84)
Graded diagnosis and treatment	486 (36.13)
Smart health care	36 (2.68)
Healthy community	24 (1.78)
Healthy China	21 (1.56)

### Stakeholder Analysis of Policy Tools

On the basis of the policy tool dimension, a stakeholder dimension was introduced for joint analysis in Table 6. The results show that the 3 types of policy tools involving the government had a relatively even distribution, with environment-based policy tools having the highest frequency of use at 64% (992/1550) and demand-based policy tools having the lowest frequency of use at 46.6% (385/827). For second-class-and-above hospitals, the proportion of the 3 types of policy tools was not significantly different—supply-based policy tools were the most frequently used, accounting for 29.4% (182/620), whereas environment-based policy tools were the least frequently used, accounting for 20.19% (313/1550). For grassroots hospitals, demand-based policy tools had the highest proportion at 20.8% (172/827), and environment-based policy tools had the lowest proportion at 13.94% (216/1550). Examining the internal composition, it was observed that, for the government, the *technical support,* *financial guarantee*, *adhere to public welfare,* *medicine supply*, *price adjustment*, and *policy promotion* tools were relatively lacking compared with other tools of the same type. For second-class-and-above hospitals and grassroots hospitals, although there were few policy items, including *financial guarantee* and *adhere to public welfare*, these 2 items should be led by the government, so a small proportion of items is understandable. However, *technical support*, *price adjustment,* and *policy promotion* were relatively insufficiently used. In addition, the classification of physicians and residents did not contain any *policy promotion* items, highlighting an existing issue.

**Table 6:** 2D distribution table of policy tools and stakeholders of the integrated health management system (n=2997).

Policy tool and secondary classification		Governments, n (%)	Second-class-and-above hospitals, n (%)	Grassroots hospitals, n (%)	Physicians, n (%)	Residents, n (%)
**Supply-based**						
	Talent construction (n=143)	53 (37.06)	46 (32.17)	36 (25.17)	8 (5.59)	N/A^a^
	Financial guarantee (n=42)	37 (88.1)	2 (4.76)	2 (4.76)	1 (2.38)	N/A
	Information support (n=225)	121 (53.78)	76 (33.78)	27 (12)	1 (0.44)	N/A
	Technical support (n=30)	15 (50)	10 (33.33)	5 (16.77)	N/A	N/A
	Rational layout (n=180)	90 (50)	48 (26.67)	40 (22.22)	2 (1.11)	N/A
	Total (n=620)	316 (50.97)	182 (29.35)	110 (17.74)	12 (1.94)	0 (0)
**Demand-based**						
	Medical insurance payment (n=94)	73 (77.66)	11 (11.7)	8 (8.51)	2 (2.13)	N/A
	Medicine supply (n=63)	35 (55.56)	15 (23.81)	12 (19.05)	1 (1.59)	N/A
	Price adjustment (n=52)	38 (73.08)	8 (15.38)	6 (11.54)	N/A	N/A
	Ability building (n=117)	43 (36.75)	40 (34.19)	33 (28.21)	1 (0.85)	N/A
	Adhere to public welfare (25)	23 (92)	1 (4)	1 (4)	N/A	N/A
	Orderly medical treatment (n=396)	121 (30.56)	118 (29.8)	99 (25)	48 (12.12)	10 (2.53)
	Demonstration project (n=80)	52 (65)	15 (18.75)	13 (16.25)	N/A	N/A
	Total (n=827)	385 (46.55)	208 (25.15)	172 (20.8)	52 (6.29)	10 (1.21)
**Environment-based**						
	Objective programming (n=273)	219 (80.22)	30 (10.99)	20 (7.33)	3 (1.1)	1 (0.37)
	Institutional building (n=371)	239 (64.42)	64 (17.25)	51 (13.75)	14 (3.77)	3 (0.81)
	Incentive and restraint (n=241)	169 (70.12)	48 (19.92)	20 (8.3)	3 (1.24)	1 (0.41)
	Policy promotion (n=56)	48 (85.71)	3 (5.36)	5 (8.93)	N/A	N/A
	Functional localization (n=351)	189 (53.85)	94 (26.78)	68 (19.37)	N/A	N/A
	Resource sharing (n=258)	128 (49.61)	74 (28.68)	52 (20.16)	4 (1.55)	N/A
	Total (n=1550)	992 (64)	313 (20.19)	216 (13.94)	24 (1.55)	5 (0.32)
Grand total (n=2997)		1693 (56.49)	703 (23.46)	498 (16.62)	88 (2.94)	15 (0.5)

^a^N/A: not applicable.

### Analysis of Level Differences

#### Analysis of the Level Differences of Policy Tools

On the basis of the policy tool dimension, the policy level dimension was introduced to analyze differences. Table 7 shows that, when considering all provinces and cities except Shanghai as a whole, the number and proportion of policies was greater than that of the central government and Shanghai, and the distribution proportion was even regarding supply-, demand-, and environment-based tools. Under the second classification of supply-based policy tools, compared with the national IHMS policy, other provinces and cities had a higher proportion of *rational layout*, *talent construction*, and *financial guarantee*, whereas Shanghai had a higher proportion of *technical support* and a lower proportion of *talent construction*. Under the second classification of demand-based policy tools, the proportion of national IHMS policies in *orderly medical treatment* and *demonstration project* was higher, whereas the proportion of *price adjustment* and *adherence to public welfare* was lower. Other provinces and cities had a higher proportion of *orderly medical treatment* and *medical insurance payment*, whereas Shanghai had a higher proportion of *ability building* and the lowest proportion of *medicine supply*. Under the second classification of environment-based policy tools, compared with the national IHMS policy, other provinces and cities had a higher proportion of *institutional building* and *objective programming*, whereas the proportion of *incentive and restraint* was slightly lower. In contrast, the proportion of *objective programming* and *institutional building* was slightly higher in Shanghai.

**Table 7:** 2D distribution table of policy tools and policy levels of the integrated health management system (n=1929).

Policy tool and secondary classification		National level, n (%)	Provincial and municipal level, n (%)	Shanghai, n (%)
**Supply-based**				
	Talent construction (n=75)	10 (13.33)	59 (78.67)	6 (8)
	Financial guarantee (n=37)	4 (10.81)	28 (75.68)	5 (13.51)
	Information support (n=127)	33 (25.98)	77 (60.63)	17 (13.39)
	Technical support (n=18)	2 (11.11)	8 (44.44)	8 (44.44)
	Rational layout (n=100)	12 (12)	76 (76)	12 (12)
	Total (n=357)	61 (17.09)	248 (69.47)	48 (13.45)
**Demand-based**				
	Medical insurance payment (n=73)	6 (8.22)	61 (84.56)	6 (8.22)
	Medicine supply (n=40)	5 (12.5)	34 (85)	1 (2.5)
	Price adjustment (n=38)	2 (5.26)	33 (86.84)	3 (7.89)
	Ability building (n=59)	8 (13.56)	39 (66.1)	12 (20.34)
	Adhere to public welfare (n=24)	4 (16.67)	17 (70.83)	3 (12.5)
	Orderly medical treatment (n=197)	53 (26.9)	131 (66.5)	13 (6.6)
	Demonstration project (n=52)	16 (30.77)	31 (59.62)	5 (9.62)
	Total (n=483)	94 (19.46)	346 (71.64)	43 (8.9)
**Environment-based**				
	Objective programming (n=223)	21 (9.42)	173 (77.58)	29 (13)
	Institutional building (n=253)	38 (15.02)	193 (76.28)	22 (8.7)
	Incentive and restraint (n=187)	41 (21.93)	120 (64.17)	26 (13.9)
	Policy promotion (n=49)	6 (12.24)	36 (73.47)	7 (14.29)
	Functional localization (n=241)	64 (26.56)	150 (62.24)	27 (11.2)
	Resource sharing (n=136)	26 (19.12)	92 (67.65)	18 (13.24)
	Total (n=1089)	196 (18)	764 (70.16)	129 (11.85)
Grand total (n=1929)		351 (18.2)	1358 (70.4)	220 (11.4)

#### Analysis of the Level Differences of Policy Orientation

Table 8 displays the distribution of IHMS policy orientations and policy levels. Across all policy levels, IHMS policies contained more content on graded diagnosis and treatment and medical association but less content on the smart health care, healthy community, and healthy China orientations. However, policies issued by Shanghai had a higher proportion of relevant content on healthy community (9/158, 5.7%) and healthy China (4/158, 2.5%) compared with national IHMS policies on these 2 orientations. The proportion of relevant content on smart health care in the national IHMS policies was higher (10/275, 3.6%) than that in policies issued by Shanghai and other provinces and cities. This indicates that Shanghai, as a pioneer city in the implementation of IHMS policies, has played a leading role in the construction of healthy communities and healthy China, but more efforts are needed to strengthen the construction of smart health care.

**Table 8:** 2D distribution table of policy orientations and policy levels of the integrated health management system.

Policy orientation	National level (n=275), n (%)	Shanghai (n=158), n (%)	Provincial and municipal level (n=912), n (%)
Graded diagnosis and treatment	120 (43.6)	49 (31)	317 (34.8)
Healthy community	9 (3.3)	9 (5.7)	6 (0.7)
Healthy China	6 (2.2)	4 (2.5)	11 (1.2)
Medical association	130 (47.3)	92 (58.2)	556 (61)
Smart health care	10 (3.6)	4 (2.5)	22 (2.4)

### Analysis of Nature Differences

#### Analysis of the Nature Differences of Policy Tools

The dimension of policy nature was introduced to analyze the differences in addition to the dimension of policy tools. As shown in Table 9, demand-based policy tools accounted for 55.9% (270/483) of the planned IHMS policy items, and environment-based policy tools accounted for 53.63% (584/1089) of the implementation details. Under the second classification of supply-based policy tools, the implementation rules had a higher proportion of *technical support* and *rational layout* but a lower proportion of *talent construction* and *financial guarantee* compared with the planned IHMS policy. Under the second classification of demand-based policy tools, the implementation rules had a higher proportion of *demonstration project*, whereas the proportion of *orderly medical treatment* in the planned IHMS policy items was lower than that in the implementation rules. Under the secondary classification of environment-based policy tools, the implementation rules had a higher proportion of *incentive and restraint*, *functional localization*, and *objective programming* but a lower proportion of *policy promotion* and *resource sharing* compared with the planned IHMS policy.

**Table 9:** 2D distribution table of policy tools and policy nature of the integrated health management system (n=1929).

Policy tool and secondary classification			Planning policy, n (%)		Detailed regulations, n (%)
**Supply-based**					
	Talent construction (n=75)	47 (63)		28 (37)	
	Financial guarantee (n=37)	21 (57)		16 (43)	
	Information support (n=127)	58 (45.7)		69 (54.3)	
	Technical support (n=18)	9 (50)		9 (50)	
	Rational layout (n=100)	46 (46)		54 (54)	
	Total (n=357)	181 (50.7)		176 (49.3)	
**Demand-based**					
	Medical insurance payment (n=73)	45 (62)		28 (38)	
	Medicine supply (n=40)	20 (50)		20 (50)	
	Price adjustment (n=38)	24 (63)		14 (37)	
	Ability building (n=59)	40 (68)		19 (32)	
	Adhere to public welfare (n=24)	14 (58)		10 (42)	
	Orderly medical treatment (n=197)	112 (56.9)		85 (43.1)	
	Demonstration project (n=52)	15 (29)		37 (71)	
	Total (n=483)	270 (55.9)		213 (44.1)	
**Environment-based**					
	Objective programming (n=223)	101 (45.3)		122 (54.7)	
	Institutional building (n=253)	123 (48.6)		130 (51.4)	
	Incentive and restraint (n=187)	67 (35.8)		120 (64.2)	
	Policy promotion (n=49)	29 (59)		20 (41)	
	Functional localization (n=241)	108 (44.8)		133 (55.2)	
	Resource sharing (n=136)	77 (56.6)		59 (43.4)	
	Total (n=1089)	505 (46.37)		584 (53.63)	
Grand total (n=1929)			956 (49.56)		973 (50.44)

#### Analysis of the Nature Differences of Policy Orientation

Table 10 shows the distribution of IHMS policy orientation and nature. The proportion of planned IHMS policies related to healthy China was relatively high (11/674, 1.6%), whereas the content related to implementing IHMS policies on healthy community and smart health care was relatively high (16/671, 2.4% and 21/671, 3.1%, respectively).

**Table 10:** 2D distribution table of policy orientation and policy nature of the integrated health management system.

Policy orientation	Planning policy (n=674), n (%)	Detailed regulations (n=671), n (%)
Graded diagnosis and treatment	345 (51.2)	141 (21)
Healthy community	8 (1.2)	16 (2.4)
Healthy China	11 (1.6)	10 (1.5)
Medical association	295 (43.8)	483 (72)
Smart health care	15 (2.2)	21 (3.1)

### Analysis of Time Evolution

#### Analysis of the Time Evolution of Policy Tools

On the basis of the dimension of policy tools, the release time of policies was introduced for the analysis of evolution. As shown in Table 11, the distribution of the number of IHMS policy texts in each year exhibited a trend of initially increasing and then decreasing, reaching its peak in 2017 and declining in the last 2 years.

Regarding supply-based policy tools, the number of policies related to *technical support* was the lowest, but it increased in the last 2 years. The change trend in the number of other secondary tools was more consistent with the overall trend, that is, they first increased and then decreased, with the lack of *financial guarantee* being more prominent in the last 2 years. Demand-based policy tools were used less frequently from 2019 to 2021, with only a small number of policies remaining related to *ability building* and *orderly medical treatment*. The use of environment-based policy tools was relatively high, but the number of secondary tools also dropped significantly since 2017. In the last 2 years, except for *functional localization*, a certain number of policies were maintained, and the frequency of use of other tools was low, especially *policy promotion*.

**Table 11:** 2D distribution table of policy tools and release time of the integrated health management system (n=1929).

Policy tool and secondary classification		2015 and before, n (%)	2016, n (%)	2017, n (%)	2018, n (%)	2019, n (%)	2020, n (%)	2021, n (%)
**Supply-based**								
	Talent construction (n=75)	13 (17)	12 (16)	29 (39)	8 (11)	8 (11)	1 (1)	4 (5)
	Financial guarantee (n=37)	2 (5)	6 (16)	20 (54)	1 (3)	6 (16)	2 (5)	N/A^a^
	Information support (n=127)	11 (8.7)	19 (15)	39 (30.7)	22 (17.3)	19 (15)	14 (11)	3 (2.4)
	Technical support (n=18)	1 (6)	N/A	9 (50)	2 (11)	1 (6)	2 (11)	3 (17)
	Rational layout (n=100)	9 (9)	2 (2)	71 (71)	3 (3)	5 (5)	6 (6)	4 (4)
	Total (n=357)	36 (10.1)	39 (10.9)	168 (47.1)	36 (10.1)	39 (10.9)	25 (7)	14 (3.9)
**Demand-based**								
	Medical insurance payment (n=73)	9 (12)	17 (23)	35 (48)	6 (8)	6 (8)	N/A	N/A
	Medicine supply (n=40)	6 (15)	5 (13)	21 (53)	6 (15)	1 (3)	1 (3)	N/A
	Price adjustment (n=38)	6 (16)	15 (39)	15 (39)	1 (3)	1 (3)	N/A	N/A
	Ability building (n=59)	9 (15)	13 (22)	17 (29)	5 (8)	10 (17)	2 (3)	3 (5)
	Adhere to public welfare (n=24)	1 (4)	2 (8)	17 (71)	3 (13)	N/A	1 (4)	N/A
	Orderly medical treatment (n=197)	43 (21.8)	54 (27.4)	71 (36)	9 (4.6)	15 (7.6)	3 (1.5)	2 (1)
	Demonstration project (n=52)	6 (12)	10 (19)	21 (40)	2 (4)	13 (25)	N/A	N/A
	Total (n=483)	80 (16.6)	116 (24)	197 (40.8)	32 (6.6)	46 (9.5)	7 (1.4)	5 (1)
**Environment-based**								
	Objective programming (n=223)	24 (10.8)	29 (13)	132 (59.2)	21 (9.4)	9 (4)	5 (2.2)	3 (1.3)
	Institutional building (n=253)	25 (9.9)	42 (16.6)	127 (50.2)	33 (13)	10 (4)	12 (4.7)	4 (1.6)
	Incentive and restraint (n=187)	15 (8)	23 (12.3)	72 (38.5)	56 (29.9)	9 (4.8)	9 (4.8)	3 (1.6)
	Policy promotion (n=49)	9 (18)	14 (29)	19 (39)	2 (4)	3 (6)	1 (2)	1 (2)
	Functional localization (n=241)	23 (9.5)	39 (16.2)	103 (42.7)	19 (7.9)	28 (11.6)	17 (7.1)	12 (5)
	Resource sharing (n=136)	16 (11.8)	16 (11.8)	72 (52.9)	7 (5.1)	11 (8.1)	10 (7.4)	4 (2.9)
	Total (n=1089)	112 (10.28)	163 (15.15)	525 (48.21)	138 (12.67)	70 (6.43)	54 (4.96)	27 (2.48)
Grand total (n=1929)		228 (11.82)	318 (16.49)	890 (46.14)	206 (10.68)	155 (8.04)	86 (4.46)	46 (2.38)

^a^N/A: not applicable.

#### Analysis of the Time Evolution of Policy Orientation

Table 12 presents the distribution of IHMS policy orientation and release time. Between 2014 and 2021, the proportion of graded diagnosis and treatment, healthy China, medical association, and smart health care increased initially and then decreased, with the highest proportions occurring in 2016, 2019, 2017, and 2018, respectively. However, the proportion of healthy community guidance–related content showed a certain growth trend, particularly in the last 2 years. This suggests that the change order of IHMS policy orientation is roughly as follows: graded diagnosis and treatment, medical association, smart health care, healthy China, and healthy community. This indicates that the government is increasingly prioritizing the all-around health needs of the people with *health as the goal*, promoting the construction of the IHMS, and taking the community as an essential starting point.

**Figure 4:**
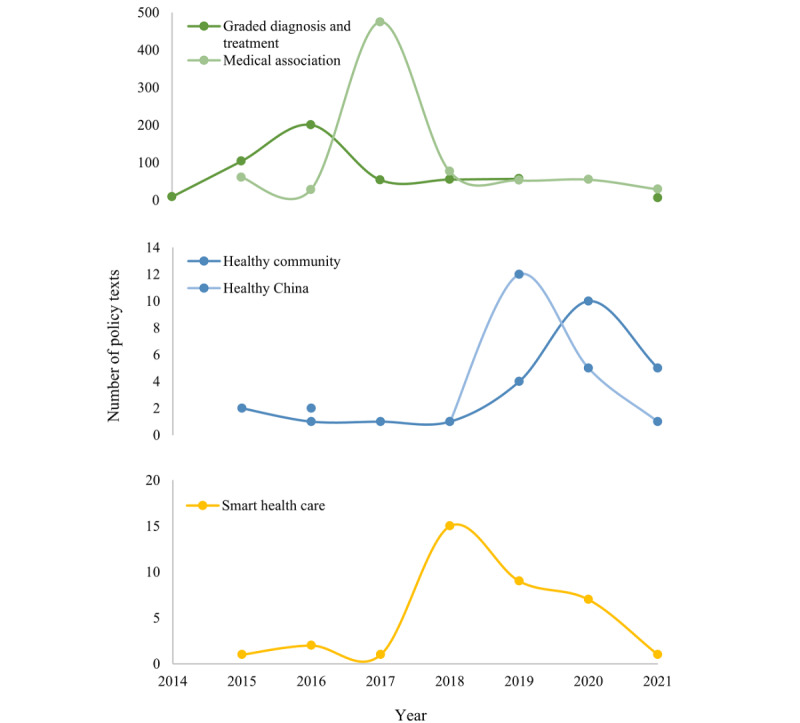
Change chart for the quantity of policies on each policy orientation of the integrated health management system under the time axis.

Figure 4 presents the changes in the 5 types of policies more intuitively. From 2014 to 2016, policies on graded diagnosis and treatment developed rapidly and then gradually slowed down, with a short stagnation in 2020. Policies on medical association started in 2015 and, after a short decline in 2016, increased in 2017, and the development speed gradually slowed down. Policies on healthy community developed steadily since 2015 and reached a small peak in 2020. Policies on healthy China began in 2016 but experienced a blank period in 2017. They developed rapidly from 2018 to 2019 and then gradually declined. Smart health care policies developed rapidly from 2015 to 2018 and then declined year by year.

**Table 12:** 2D distribution table of policy orientations and release time of the integrated health management system (n=1345).

Policy orientation	2014 (n=9), n (%)	2015 (n=168), n (%)	2016 (n=234), n (%)	2017 (n=531), n (%)	2018 (n=149), n (%)	2019 (n=135), n (%)	2020 (n=77), n (%)	2021 (n=42), n (%)	Total, n (%)
Graded diagnosis and treatment	9 (100)	104 (61.9)	201 (85.9)	54 (10.2)	55 (36.9)	57 (42.2)	N/A^a^	6 (14.3)	486 (36.1)
Healthy community	N/A	2 (1.2)	1 (0.4)	1 (0.2)	1 (0.7)	4 (3)	10 (13)	5 (11.9)	24 (1.8)
Healthy China	N/A	N/A	2 (0.9)	N/A	1 (0.7)	12 (8.9)	5 (6.5)	1 (2.4)	21 (1.6)
Medical association	N/A	61 (36.3)	28 (12)	475 (89.5)	77 (51.7)	53 (39.3)	55 (71.4)	29 (69)	778 (57.8)
Smart health care	N/A	1 (0.6)	2 (1.2)	1 (0.2)	15 (10.1)	9 (6.7)	7 (9.1)	1 (2.4)	36 (2.7)

^a^N/A: not applicable.

## Discussion

### Principal Findings

#### Essential Features

This study found that China’s IHMS policy showed more distinctive features under the 3 dimensions of release time, policy type, and policy level. In terms of release time, there was an abrupt increase before 2017, followed by a decline from 2018 to 2019. They remained at a high level, whereas the last 2 years saw a significant downward trend (the effect of the statistical period was not excluded). In terms of policy nature, the number of China’s IHMS policy texts on planning and implementation regulations was comparable, but the combination of the time and type of policy documents released shows that there were more general documents such as outlines and plans before 2017, more implementation documents such as opinions and notices from 2018 to 2019, and more prescriptive documents such as programs and methods from 2020 to the present, suggesting that China’s IHMS policy construction has gradually improved from top-level design to work requirements. In terms of policy level, IHMS policy texts at the provincial level were the most numerous, followed by the national and other municipal levels and, finally, Shanghai, with the number of policy texts across all of them exceeding 150, indicating that each local government is following the central government’s promotion of health consortium construction at a faster pace and with a higher degree of importance.

#### Coordination of Policy Tools and Stakeholders

##### The Proportion of Policy Tools Used and the Balance of Internal Structure Still Need to Be Improved

To optimize the proportion of policy tools, we need to focus on increasing the use of supply-based policy tools. This can be achieved by expanding the talent team, increasing scientific and technological information support, providing financial support, introducing advanced diagnosis and treatment technologies, and using public services to improve the coverage of medical services in hospitals at all levels. Demand-based policy tools also need to be further developed to fully use the economic leverage effect of medical insurance funds. This can be done by speeding up the establishment of a unified medical insurance settlement system and medical price system and supporting social medical institutions to join the system construction and expand multilevel and diversified services. It is important to note that, although environment-based policy tools are used more frequently, their limited permeability to the construction of the IHMS requires the government to reduce the proportion of some planning tools. By doing so, we can achieve a more balanced internal structure of policy tools in the construction of the IHMS.

The internal structure of policy tools requires further optimization. Supply-based policy tools such as *technical support* and *financial guarantee* are underused, and demand-based policy tools such as *adherence to public welfare*, *price adjustment*, and *medicine supply* need to be strengthened. Environment-based policy tools such as *functional localization*, *institutional building*, and *objective programming* are more closely related to IHMS, but *policy promotion* is relatively less used. First, the government needs to increase financial input, optimize the investment direction and structure, and pay attention to the proportion of the application of financial support tools. Medical institutions should upgrade their key technologies and treatment methods, especially for chronic and major diseases. Second, the government should provide tax deductions or preferential treatment for different modes of IHMS, establish a dynamic adjustment mechanism for medical service prices, and control drug prices and supply. Finally, the government should strengthen policy propaganda, use new media to popularize and publicize the IHMS, and provide training for medical staff and health education for residents. The government should properly control the use of tools with strong intervention; give relevant units autonomy; and mobilize participants’ enthusiasm and sense of responsibility through performance incentives, taxation, and subsidies.

##### The IHMS Policy Still Needs to Balance the Attention Paid to All Stakeholders

The current IHMS policy tools show a greater focus on the government and pay less attention to hospitals, physicians, and residents at all levels, particularly grassroots hospitals, physicians, and residents. Therefore, adjustments must be made to the use ratio of policy tools, with a greater emphasis on supporting grassroots hospitals to enhance their service capacity and efficiency through talent and technology support. Second-class-and-above hospitals should also collaborate with grassroots hospitals to carry out exchange projects and establish a connection. The government should also urge all hospitals to accelerate the implementation of the multipoint practice system of physicians and the contract system of family physicians, build a multilevel team of physicians, and establish a physician cooperation mechanism to improve the ability of primary medical services through counterpart assistance and business guidance. Furthermore, the government must improve residents’ awareness and participation in the policy; guide residents in need to experience the medical treatment process under the IHMS operating system; and encourage them to actively participate in health knowledge forums, IHMS medical treatment exchange meetings, and other activities. Finally, more third-party health institutions such as medical insurance departments and rehabilitation centers should be included so that residents can enjoy health services and protection throughout their life cycle.

#### Level Differences

##### Level Differences of Policy Tools

Governments at different levels exhibited similarities and differences in their use of policy tools for IHMS. Environment-based policy instruments were most frequently used at each level of IHMS policy, whereas supply-based policy tools were least commonly used at the national level and in other provincial and local IHMS policies. Demand-based policy tools were least prevalent in Shanghai. Specifically, the proportion of *rational layout*, *talent construction*, *institutional building*, and *objective programming* in the IHMS policies issued by the central government was relatively low. The proportion of *demonstration project*, *resource sharing*, and *incentive and restraint* in the IHMS policies issued by other provinces and cities was relatively low. Finally, *talent construction* and *institutional building* were rarely used in the IHMS policies in Shanghai. Therefore, the central government should make use of low-frequency policy tools more frequently, whereas other governments at all levels should respond to the state’s initiatives and incorporate local characteristics to enhance the current structure’s rationality.

Local authorities can take into account local characteristics and respond to national initiatives by considering the following features to improve the effectiveness of the current structure:

1. Local medical resources: as medical resources are distributed differently across regions, authorities can tailor their policies to local circumstances considering the availability and workforce of local medical facilities. For example, where medical resources are in short supply, the government can introduce incentive schemes to attract medical practitioners and other professionals to work in that region.
2. Demand for medical services: given the differences in the demographic structure, disease spectrum, and health requirements of different regions, local governments can develop targeted policies that cater to specific local conditions. For instance, in areas with a significant aging population, the government can offer more support for older adult care, long-term care, and management of geriatric diseases.
3. Local culture and community characteristics: each region has its own cultural and community features, which local governments can include in smart health care and healthy community policies to improve residents’ sense of identification and engagement. For instance, health-related promotional activities can coincide with special folk festivals to inspire local participation in diverse activities focused on health promotion.

##### Level Differences of Policy Orientation

Different levels of IHMS policies have varying emphases on different aspects. The national IHMS policy places greater emphasis on smart health care (including digital health), whereas Shanghai has made a better effort to create healthy communities and promote a healthy China. To achieve a balanced growth, local governments should accurately understand the central government’s primary policy orientation, catch up with the promotion of smart health care policy, and simultaneously increase their efforts to support the construction of healthy communities and healthy China. This will help create a more comprehensive and well-rounded IHMS policy system that can better meet the needs of the people.

Local governments can do the following to strengthen support for smart health care and healthy community:

1. Healthy community construction: local governments can provide financial and resource support to develop healthy communities that offer convenient medical and health services to residents, for instance, set up health consultation centers or community medical stations or organizing regular health checkup activities.
2. Smart medical technology promotion: local governments can organize training and promotion activities to improve medical staff’s knowledge of and ability to use smart medical technology and promote the adoption of advanced IT by medical institutions to improve the efficiency of diagnosis and treatment and the quality of service. For example, promoting telemedicine and electronic medical record systems could improve the use of medical resources.
3. Health education and publicity: local governments can increase their efforts in health education and publicity to enhance residents’ health awareness and literacy. They can achieve this goal by conducting health information lectures, publishing health promotion materials, and organizing health activities to foster healthy behaviors among residents.

#### Nature Differences

##### Differences in the Nature of Policy Tools

Demand-based policy tools constituted a relatively high proportion of the planned IHMS policy, whereas environment-based policy tools constituted a relatively high proportion of the implementation details. However, *technical support*, *adherence to public welfare*, and *policy promotion* were not used very often. Conversely, the planned IHMS policy had a very low use of *demonstration projects* and *incentive and restraint* tools, whereas the comprehensive IHMS policy used fewer mechanisms such as *talent construction*, *financial guarantee*, and *medical insurance payment.* Therefore, the government should prioritize the implementation plan and important measures such as medical insurance and price reform and strengthen the planning and design of pilot systems, assessments, and incentive systems.

Using health insurance reform as an example, the government can take the following steps to strengthen planning and design to ensure smooth implementation and effective outcomes:

1. Creating a detailed implementation program: the government must develop a detailed implementation program that outlines the specific content, timeline, and departments responsible for each policy measure. For example, the mechanism for adjusting drug prices and payment for medical services should be clarified to ensure that the various reform initiatives can be implemented in an orderly manner.
2. Assessing risk factors: during the planning and design process, the government should fully consider potential risks and challenges and create responsive strategies. As an illustration, when adjusting health insurance payments that could provoke protests from some hospitals, the government should anticipate this and create solutions beforehand.
3. Monitoring and evaluation mechanism: establish a robust monitoring and evaluation mechanism to regularly assess the effectiveness of the health care reform implementation so that problems can be identified and policies can be adjusted in a responsive manner. The government can set up a special monitoring team to report regularly on the progress and effectiveness of the reform, thereby increasing transparency and accountability.

##### Differences in the Nature of Policy Orientation

Both the planned and detailed implementation rules for IHMS policies prioritize medical association and graded diagnosis and treatment while placing less emphasis on smart health care, healthy community, and healthy China. However, in contrast, the planned policies give more importance to healthy China, whereas the implementation rules concentrate more on smart health care and healthy community. To strengthen the implementation of the healthy China policy, the government should supplement it with detailed policies such as implementation plans and methods. For the smart health care and healthy community policies, which have stronger operational attributes, the government should develop comprehensive plans to improve the system of IHMS policies.

For the operational attributes of the smart health care and healthy community policies, the government should develop a comprehensive plan to improve its policy system, including the following:

1. Technical support and standards: the government should establish a unified technical support system for smart health care and healthy communities. Relevant technical standards and specifications should be developed to ensure the consistency and interoperability of implementation across regions.
2. Data management and privacy protection: the government should formulate sound data management policies and privacy protection measures to ensure that the personal health data involved in smart health care and healthy communities are properly managed and protected to prevent data leakage and abuse.
3. Cooperation mechanisms and coordination bodies: the government can develop a cross-sectoral and interdisciplinary cooperation mechanism and set up a special body to facilitate the implementation and coordination of smart health care and healthy community policies. For example, a special committee can be established comprising relevant departments and experts to be responsible for policy formulation, resource allocation, and coordination of promotion activities.

#### Time Evolution

##### Time Evolution of Policy Tools

Generally speaking, from 2014 to 2021, there was a trend of rising and falling for various policy tools. Among them, environment-based policy tools were used more frequently, whereas demand-based policy tools were relatively insufficiently used in the past 3 years. In the past 2 years, there has been a shortage of *financial guarantee*, *medical insurance payment*, *medicine supply*, *price adjustment*, *adherence to public welfare*, *demonstration projects*, and *policy promotion*. Although the amount of *technical support* and *capacity building* was small, it has shown an increasing trend in the last 2 years, whereas the number of other policy tools was small and showed a declining trend. To address this issue, the government should first increase the application of demand-based policy tools and then focus on improving the shortcomings of supply-based and environment-based policy tools.

##### Time Evolution of Policy Orientation

From 2014 to 2021, the IHMS policy orientation shifted from graded diagnosis and treatment to medical association to smart health care and, finally, to healthy China and healthy community. Graded diagnosis and treatment and medical association policies focus on disease diagnosis and treatment, whereas healthy community and healthy China policies focus on health management and smart health care emphasizes the technical means of IHMS operation. We believe that the development of the IHMS policy system can be divided into 3 stages: the *disease-centered period* (2014-2017), dominated by graded diagnosis and treatment and medical association policies; the *electronic health technology development period* (2017-2019), during which smart health care policies were vigorously developed; and the *health-centered period* (2018-2021), which mainly promoted the healthy China and healthy community policies and tended to lean toward healthy community policies. As a result, the IHMS policy system took smart health care policy as a bridge to realize the transformation from *treatment-centered* to *health-centered.* The government should increase multidimensional horizontal integration exploration with the community as the starting point, give primary physicians a pivotal role, and provide people with all-around and full-cycle health services by using electronic health technology.

### Contributions and Limitations

#### Theoretical Contributions

This study makes 3 theoretical contributions.

First, we expanded the object of policy research and developed a comprehensive analysis. The primary contribution of this study to the field of policy analysis is that we have broadened the object of study; conducted in-depth research on multiple types of policies; and constructed an IHMS policy system with common goals, multiple subjects, and complex interactions. This is different from previous studies (eg, Yang et al [26] and Kaló et al [27]) in that we took the concepts and connotations of IHMS as the basis of our study, selected 5 categories of policies (graded diagnosis and treatment, medical association, healthy China, healthy community, and smart health care) to study, and used content analysis to comprehensively understand their characteristics. This is distinctive in that it integrates different policies into a holistic framework and explores their interrelationships. This comprehensive analytical approach integrates quantitative statistics and qualitative analysis and allowed us to examine the characteristics and determinants of IHMS policies from multiple dimensions, making the findings more comprehensive and richer. This comprehensive analytical approach allowed us to gain an in-depth understanding of the interrelationships between the dimensions, thus providing more accurate research conclusions. With the data support of quantitative statistics, we enhanced the credibility of our study and also enriched its content, making it more valuable in both theory and practice.

Second, we enriched the level of policy analysis and provided decision support. The second contribution of this study is that we included different levels of policy factors in our policy analysis, in particular by considering local policies. This differs from previous studies (eg, Ebrahim et al [28] and Wu et al [29]) that focused on only one level of policy analysis as we showed the diffusion, speed, and impact of policies by comparing the similarities and differences between national and local policies in other dimensions, which helped identify regional characteristics for future research. This is characterized by the fact that we extended the influence of policies to different levels, thus providing a more comprehensive understanding of the multidimensional effects of policies. This contributes to a better understanding of the complexity of policies and provides a more accurate reference and support for policy makers. By analyzing the relationships between the dimensions in depth, we drew conclusions that provide practical guidance for policy formulation and implementation. The value of this practical application not only makes our findings meaningful in the academic field but also provides useful references and decision support for policy makers.

Third, we improved the policy analysis framework to achieve a systematic analysis. The third contribution is that we improved the existing policy analysis framework to make it more applicable to policy cluster or policy network analysis. Compared with previous studies that proposed 2D (Xie and Tian [30]), 3D (Shen et al [31]), or 4D (Yang et al [26]) frameworks, we proposed a 6D policy analysis framework that includes the dimensions of policy tools, stakeholders, policy level, policy nature, policy orientation, and release time and takes policy orientation and policy tool as the core dimensions, focusing on their hierarchical and temporal differences. It is characterized by a more comprehensive analytical framework covering multiple dimensions of consideration. This systematic approach to analysis provided us with a more comprehensive and multidimensional perspective, which helped us gain insights into the characteristics and influences of policies. This improvement enabled us to better grasp the relationships between the dimensions and, thus, gain a more comprehensive understanding of the nature of the policy. This systematic analysis added depth and breadth to our study and helped understand the multilevel and multidimensional characteristics of policy in a more comprehensive way, thus providing a more valuable reference base for future policy formulation and implementation.

#### Practical Enlightenment

These results have practical value in 2 respects. First, they aid central policy makers in evaluating the macrostructural design of the IHMS to optimize it. The results can also assist local policy makers in better understanding the central authorities’ emphasized key contents and in improving common issues, leading to IHMS policy promotion based on local conditions. Second, the results can assist IHMS members in comprehending their respective responsibilities and work priorities and taking measures to enhance their division of labor and cooperation.

#### Limitations and Future Work

This study has some limitations. First, it does not provide a more in-depth analysis combined with regional division, which limits the regional characteristics obtained. Second, frequency or frequency-based statistics are only applicable to text corpora that do not reach the level of big data and cannot effectively mine the hidden information in policy texts. Third, owing to time and resource constraints, we did not use machine learning models for implicit semantic analysis, and some text-mining algorithms could be incorporated to deal with large-scale public policy texts in future research.

### Conclusions

China’s IHMS policy has specified the development objectives and core contents of the health consortium and facilitated the promotion of the rational allocation of health resources in China. This paper presents a comprehensive analysis of China’s IHMS policy from 6 perspectives, namely, policy level, policy nature, release time, policy tools, stakeholders, and policy orientation, using the PRISMA guidelines and content analysis to reveal the changes and hierarchical differences in the structure of different types of policies and policy tools in the system and provide some possible lessons for future policy formulation and optimization.

## Appendix

Multimedia Appendix 1 Specifications of China’s integrated health management system policy.

Multimedia Appendix 2 Explanation of the 6D framework.

Multimedia Appendix 3 The PRISMA (Preferred Reporting Items for Systematic Reviews and Meta-Analyses) checklist.

Multimedia Appendix 4 Detailed information on the 152 China integrated health management system policy documents collected.

Multimedia Appendix 5 Classifications of the policy tools and stakeholders of the integrated health management system.

Multimedia Appendix 6 Specific steps for coding and manual labeling.

## Abbreviations

IHMS: integrated health management system
PRISMA: Preferred Reporting Items for Systematic Reviews and Meta-Analyses

## Data Availability

The data sets generated and analyzed during this study are available from the corresponding author upon reasonable request.
