# Supplementary material for: Understanding the Integrated Health Management System Policy in China From Multiple Perspectives: Systematic Review and Content Analysis
Source: J Med Internet Res. 2024 Jan 24;26:e47197. doi: 10.2196/47197 (PMC10851112; doi:10.2196/47197)
Supplement: Multimedia Appendix 1 [file jmir_v26i1e47197_app1.docx]

Specifications of China's Integrated Healthcare Management System policy^[[1]](#endnote-1)^

China's Integrated Healthcare Management System (IHMS) policy covers a number of oriented policies, including:

- Healthy China policy: focus on improving the health of the whole population, emphasize prevention first, strengthen primary medical services, and promote health education and health promotion.
- Graded diagnosis and treatment policy: aim to establish a tiered medical system, guide patients to seek medical care close to home, reduce the pressure on large hospitals and improve the level of primary medical care.
- Medical association policy: promote cooperation and collaboration among hospitals, form a consortium to share medical resources, and improve the overall efficiency and quality of medical services.
- Healthy community (smart community) policy: build a smart community medical service platform to provide convenient health management and medical services for residents.
- Smart healthcare policy: promote the construction of medical information, and use information and communication technologies to improve the efficiency of medical services and the accuracy of diagnosis. An important component and key application is digital health. It makes use of tools and technologies such as electronic health records, telemedicine, health data monitoring, artificial intelligence and machine learning to achieve innovative services such as medical information collection and processing, telemedicine and m-health, personalized and precision medicine, medical management and quality control, patient education and health management.

1. This is a Multimedia Appendix to a full manuscript published in the J Med Internet Res. For full copyright and citation information see http://dx.doi.org/10.2196/jmir.47197. [↑](#endnote-ref-1)
