# Supplementary material for: Understanding the Integrated Health Management System Policy in China From Multiple Perspectives: Systematic Review and Content Analysis
Source: J Med Internet Res. 2024 Jan 24;26:e47197. doi: 10.2196/47197 (PMC10851112; doi:10.2196/47197)
Supplement: Multimedia Appendix 2 [file jmir_v26i1e47197_app2.docx]

Explanation of the Six-dimensional Framework^[[1]](#endnote-1)^

We build a six-dimensional framework as described below to provide a comprehensive analysis of China's Integrated Healthcare Management System (IHMS) policy:

- Policy level: Policy level reflects the importance of the policy and the strength of its implementation. They are categorized into national, provincial and municipal levels, with special attention to certain pilot cities such as Shanghai. Policies at different levels may involve different policy content and specific measures.
- Policy nature: Policy nature refers to whether the policy is more of a planning guideline, favoring top-level design, or a more specific implementation rule. Policies are divided into planning policies and implementing regulations. Policies of different types may have different impacts on the development and implementation of health networks.
- Release time: Release time reflects the trend of the policy. Coding the time of policy release in annual units allows us to track the temporal evolution of the policy and understand the historical changes of the policy.
- Policy tools: Policy tools are the specific means of implementation and are classified into supply-based, demand-based and environment-based tools. Different types of tools may affect the progress and effectiveness of the IHMS policy.
- Stakeholders: Health consortiums involve multiple stakeholders, including the government, primary hospitals, secondary and higher hospitals, doctors and residents. These stakeholders play different roles and have an impact on policy implementation.
- Policy orientation: Policy orientation is the five types of policy that make up China's IHMS policy: graded diagnosis and treatment policy, medical association policy, healthy China policy, healthy community policy and smart healthcare policy. The setting of policy orientation determines the goal and direction of the whole policy system.

1. This is a Multimedia Appendix to a full manuscript published in the J Med Internet Res. For full copyright and citation information see http://dx.doi.org/10.2196/jmir.47197. [↑](#endnote-ref-1)
