# Supplementary material for: Understanding the Integrated Health Management System Policy in China From Multiple Perspectives: Systematic Review and Content Analysis
Source: J Med Internet Res. 2024 Jan 24;26:e47197. doi: 10.2196/47197 (PMC10851112; doi:10.2196/47197)
Supplement: Multimedia Appendix 5 [file jmir_v26i1e47197_app5.docx]

Classifications of the Policy Tools and Stakeholders of IHMS^[[1]](#endnote-1)^

**Table S1.** Classification of the policy tools of IHMS.

| Policy Tools | Secondary classification | Interpretation |
| --- | --- | --- |
| Supply-based | Talent construction | Training, education, interaction and exchange of medical service personnel in the IHMS to improve their service ability. |
|  | Financial guarantee | Governments provide financial support and corresponding subsidies to the personnel and development stages of relevant institutions of the IHMS. |
|  | Information support | Perfection of information platform in the IHMS and construction of information integration |
|  | Technical support | It involves a series of measures to improve the key technology of disease diagnosis and treatment and clinical service ability. |
|  | Rational layout | Governments rationally allocates the resources of medical institutions to form an efficient medical service system. |
| Demand-based | Medical insurance payment | Governments promote the reform of medical insurance payment method in the IHMS. |
|  | Medicine supply | Unified procurement and distribution of medicines and consumables within medical groups and county medical communities. |
|  | Price adjustment | Governments promote the reform of medical service price in the IHMS. |
|  | Ability building | Comprehensive capacity building of various medical institutions in terms of specialty, service and diagnosis and treatment. |
|  | Adhere to public welfare | Effectively safeguard and guarantee the public welfare of basic medical and health undertakings to facilitate the people and benefit the masses. |
|  | Orderly medical treatment | The process of IHMS service, such as: standardizing the two-way referral mechanism and standards, perfecting the family doctor contract system, etc. |
|  | Demonstration project | Carry out the pilot project of IHMS, sum up the pilot experience, and promote it continuously. |
| Environment-based | Objective programming | Strengthen the top-level design of the government, standardize and guide the construction of IHMS and make overall planning. |
|  | Institutional building | Governments shall establish various mechanisms for the effective operation of the IHMS and manage the daily service and quality of the IHMS. |
|  | Incentive and restraint | Including supervision and assessment, performance evaluation, salary system, etc. |
|  | Policy promotion | Publicize the IHMS policy through different publicity methods, and reasonably guide public opinion. |
|  | Functional localization | Clarify the responsibilities and functions of each participating unit, and strengthen the division of labor and cooperation with departments. |
|  | Resource sharing | The sharing of interests, information, etc. also includes the sharing of resources between different regions and different types. |

**Table S2.** Classification of the stakeholders of IHMS.

| Stakeholders | Major responsibilities |
| --- | --- |
| Governments | Coordinate the construction of IHMS, supervise and assess the specific implementation of each unit. |
| Grassroots hospitals | Mainly responsible for publicity, information registration and follow-up. |
| Second-class and above hospitals | Check and treat the transferred patients, and provide technical training for community service personnel. |
| Doctors | Including specialists and family doctors. |
| Residents | Develop a healthy lifestyle and give priority to health and prevention. |

1. This is a Multimedia Appendix to a full manuscript published in the J Med Internet Res. For full copyright and citation information see http://dx.doi.org/10.2196/jmir.47197. [↑](#endnote-ref-1)
