# Supplementary material for: Understanding the Integrated Health Management System Policy in China From Multiple Perspectives: Systematic Review and Content Analysis
Source: J Med Internet Res. 2024 Jan 24;26:e47197. doi: 10.2196/47197 (PMC10851112; doi:10.2196/47197)
Supplement: Multimedia Appendix 6 [file jmir_v26i1e47197_app6.docx]

Specific Steps of Coding and Manual Labelling^[[1]](#endnote-1)^

To ensure that the coding and manual labelling support the credibility of the results, we propose the following steps:

- Back-to-back labelling. 1362 policy texts were divided into 3 Excel files and annotated in pairs. They independently annotated the policy instruments contained in the text and the stakeholders involved in the same Excel file according to the categorization and definition in Table 2 and Table 3. During this process, the pairs do not communicate in any way and each produces two copies of the annotation results.
- Filter the datasets with different annotation results. The annotation results from the same group of two people were put into the same Excel file, and Excel's comparison and positioning function was used to identify records with different annotations and highlight them in red. Out of a total of 1,362 policy texts, 264 records with different annotation content were found.
- Discuss the revision of the records with inconsistent annotations. Two people in the same group reread the text screened in the second step and, the comparison and positioning function of Excel was used to identify records with different annotation contents and highlight them in red. They work together to modify the labelling results to reach agreement.
- Integrate and determine the final annotation results. All the records with the same annotation results and agreed after modification were integrated into one Excel file. At the same time, seventeen meaningless records that were not considered to be directly related to the medical association were eliminated, resulting in a final sample of 1,345 validly annotated policy texts.

1. This is a Multimedia Appendix to a full manuscript published in the J Med Internet Res. For full copyright and citation information see http://dx.doi.org/10.2196/jmir.47197. [↑](#endnote-ref-1)
